# Analyses of the peripheral immunome following multiple administrations of avelumab, a human IgG1 anti-PD-L1 monoclonal antibody

**DOI:** 10.1186/s40425-017-0220-y

**Published:** 2017-02-21

**Authors:** Renee N. Donahue, Lauren M. Lepone, Italia Grenga, Caroline Jochems, Massimo Fantini, Ravi A. Madan, Christopher R. Heery, James L. Gulley, Jeffrey Schlom

**Affiliations:** 1 Laboratory of Tumor Immunology and Biology, Center for Cancer Research, National Cancer Institute, National Institutes of Health, 10 Center Drive, Room 8B09, Bethesda, MD USA; 2 Genitourinary Malignancies Branch, Center for Cancer Research, National Cancer Institute, National Institutes of Health, Bethesda, MD USA

**Keywords:** Avelumab, Anti-PD-L1, Checkpoint inhibitor, Immunotherapy, Peripheral immunome, Immune subsets, ADCC, Antibody-dependent cell-mediated cytotoxicity

## Abstract

**Background:**

Multiple anti-PD-L1/PD-1 checkpoint monoclonal antibodies (MAb) have shown clear evidence of clinical benefit. All except one have been designed or engineered to omit the possibility to mediate antibody-dependent cell-mediated cytotoxicity (ADCC) as a second potential mode of anti-tumor activity; the reason for this is the concern of lysis of PD-L1 positive immune cells. Avelumab is a fully human IgG1 MAb which has been shown in prior in vitro studies to mediate ADCC versus a range of human tumor cells, and clinical studies have demonstrated anti-tumor activity versus a range of human cancers. This study was designed to investigate the effect on immune cell subsets in the peripheral blood of cancer patients prior to and following multiple administrations of avelumab.

**Methods:**

One hundred twenty-three distinct immune cell subsets in the peripheral blood of cancer patients (*n* = 28) in a phase I trial were analyzed by flow cytometry prior to and following one, three, and nine cycles of avelumab. Changes in soluble (s) CD27 and sCD40L in plasma were also evaluated. In vitro studies were also performed to determine if avelumab would mediate ADCC of PBMC.

**Results:**

No statistically significant changes in any of the 123 immune cell subsets analyzed were observed at any dose level, or number of doses, of avelumab. Increases in the ratio of sCD27:sCD40L were observed, suggesting potential immune activation. Controlled in vitro studies also showed lysis of tumor cells by avelumab versus no lysis of PBMC from five donors.

**Conclusions:**

These studies demonstrate the lack of any significant effect on multiple immune cell subsets, even those expressing PD-L1, following multiple cycles of avelumab. These results complement prior studies showing anti-tumor effects of avelumab and comparable levels of adverse events with avelumab versus other anti-PD-1/PD-L1 MAbs. These studies provide the rationale to further exploit the potential ADCC mechanism of action of avelumab as well as other human IgG1 checkpoint inhibitors.

**Trial registration:**

ClinicalTrials.gov identifier: NCT01772004 (first received: 1/14/13; start date: January 2013) and NCT00001846 (first received date: 11/3/99; start date: August 1999).

**Electronic supplementary material:**

The online version of this article (doi:10.1186/s40425-017-0220-y) contains supplementary material, which is available to authorized users.

## Background

Immune checkpoint inhibition employing monoclonal antibodies (MAbs) directed against programmed cell death protein 1 (PD-1) or programmed cell death protein-1 ligand (PD-L1) has been a major advance in the management of selected patients in several tumor types and stages (see [1, 2] for recent reviews). The general concept is that the interaction of PD-1 on immune cells with PD-L1 on tumor cells can lead to immune cell anergy and thus the lack of anti-tumor activity; the use of either anti-PD-1 or PD-L1 MAbs is designed to block this interaction leading to tumor cell lysis. The use of a human anti-PD-L1 MAb of the IgG1 isotype could potentially add another mode of anti-tumor activity. Human IgG1 MAbs have been shown to be capable of mediating antibody-dependent cell-mediated cytotoxicity (ADCC) via the interaction of the IgG1 Fc region with its ligand on human natural killer (NK) cells. One caution in the use of this approach is that several human immune cell populations also express PD-L1, and could thus potentially also be susceptible to ADCC-mediated lysis. It is for this reason that, with one exception, all of the anti-PD-L1 MAbs in clinical studies to date were constructed as either an IgG4 isotype that cannot mediate ADCC, or an IgG1 MAb engineered to be devoid of ADCC activity; the one exception is the development of the human IgG1 anti-PD-L1 MAb avelumab (MSB0010718C). We have previously shown that avelumab can mediate ADCC in vitro using as targets a range of human tumor cell lines that express PD-L1, and that this lysis can be blocked using an anti-CD16 antibody to inhibit the interaction of CD16 on NK cells with the IgG1 Fc receptor on avelumab [3–5]. We have also shown that avelumab can mediate tumor lysis in vivo using a murine tumor model [6]. A recent study also showed that the addition of avelumab to an in vitro assay leads to enhanced antigen-specific T-cell activation [7]. A Phase I dose escalation trial (NCT01772004) and use of avelumab in multiple expansion cohorts have shown evidence of clinical benefit of avelumab in patients with thymoma, mesothelioma, non-small cell lung cancer (NSCLC), ovarian, gastric and urothelial cancer, among others [8–13]. A recent phase II study [14] also demonstrated clinical activity of avelumab in Merkel cell carcinoma. In the dose escalation trial, there were no dose-limiting toxicities (DLT) in dose levels 1, 2, and 3 (1, 3, and 10 mg/kg) and one DLT on dose level 4 (20 mg/kg) concurrent with an anti-tumor response [[11]; Heery, et al. First-in-human phase 1 dose-escalation trial of avelumab. Lancet Oncol., In press]. At the time of writing, multiple Phase III trials of avelumab are ongoing in patients with a range of tumor types and stages.

As mentioned above, one of the concerns in the use of an IgG1 anti-PD-L1 MAb is the potential effect on PD-L1 expressing immune cells. We have recently described a methodology in which 123 different immune cell subsets can be analyzed by flow cytometry using one tube of blood (approximately 10^7^ peripheral blood mononuclear cells (PBMC)). We have previously reported the relative distributions of these 123 classic and refined subsets in healthy donors vs. metastatic cancer patients [15]. In this study, we interrogated the effect of administration of different doses of avelumab on multiple peripheral immune cell subsets; the 123 immune cell subsets were analyzed prior to and following one, three and nine cycles of avelumab every 2 weeks. The effect of avelumab administration on changes in soluble CD27 (sCD27) and soluble CD40 ligand (sCD40L) in plasma was also evaluated. In controlled studies, the ability of avelumab to mediate ADCC of tumor cells vs. peripheral immune cells was conducted using NK cells as effectors from five different healthy donors and three cancer patients. Potential strategies to exploit the ADCC potential of avelumab are also discussed.

## Methods

### Cancer patients and healthy donors

Flow cytometry analysis was performed on PBMC of cancer patients who were enrolled in a first-in-human, open-label, dose-escalation and expansion Phase 1 clinical trial (NCT01772004). The National Cancer Institute Institutional Review Board approved the trial procedures and informed consent was obtained in accordance with the Declaration of Helsinki. Metastatic cancer patients with solid tumors were treated with 1, 3, 10 or 20 mg/kg of avelumab every 2 weeks [Heery, et al. First-in-human phase 1 dose-escalation trial of avelumab. Lancet Oncol., In press]. Based on PBMC availability, the flow cytometry analysis included 28 patients with 12 different types of solid tumors: adrenocortical (*n* = 2), breast (*n* = 3), chordoma (*n* = 1), gastrointestinal (GI) (*n* = 7), lung (*n* = 1), mesothelioma (*n* = 3), neuroendocrine (*n* = 1), ovarian (*n* = 1), pancreatic (*n* = 4), prostate (*n* = 1), renal cell (*n* = 3) and spindle cell (*n* = 1) cancer. Patients received 1 or 3 mg/kg (*n* = 11), 10 mg/kg (*n* = 8), or 20 mg/kg (*n* = 9) of avelumab. Based on plasma availability, the soluble factor analysis included 39 patients receiving 1 or 3 mg/kg (*n* = 15), 10 mg/kg (*n* = 13), or 20 mg/kg (*n* = 11) of avelumab. PBMC for in vitro ADCC assays were obtained from five healthy donors from the NIH Clinical Center Blood Bank (NCT00001846) and three NSCLC patients enrolled in a previously described trial before the initiation of therapy [16]. PBMC for the antibody competition assay were obtained from a patient with metastatic castration resistant prostate cancer (mCRPC) enrolled in a previously described trial before the initiation of therapy [17].

### Multicolor flow cytometry

**Fig. 1:**
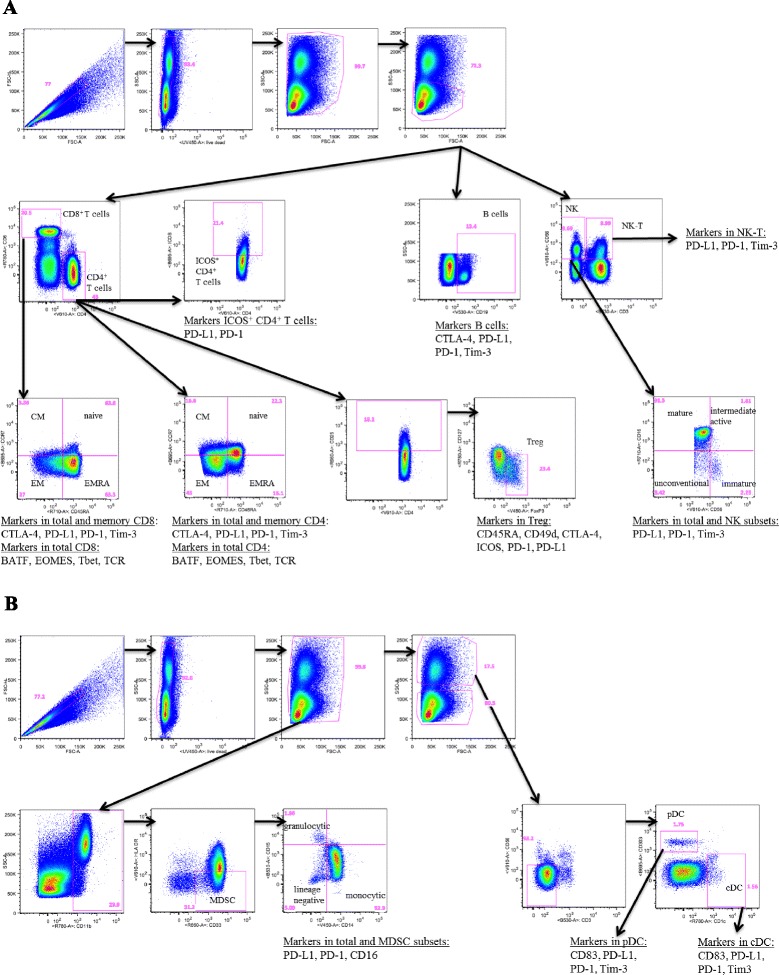
Gating strategy to identity 123 peripheral immune cell subsets. Five immune flow cytometry panels using PBMC from a cancer patient following nine cycles of avelumab were used. Classic immune cell types included CD4^+^ T cells, CD8^+^ T cells, Tregs, B cells, natural killer (NK) and NK-T cells (panel **a**), and conventional dendritic cells (cDCs), plasmacytoid DCs (pDCs) and myeloid derived suppressor cells (MDSCs) (panel **b**)

Multicolor flow cytometry was performed on frozen PBMC as previously described [15]. One vial of PBMC containing 10^7^ cells was thawed per cancer patient prior to therapy (*n* = 28) and 2 weeks after patients were administered the first cycle (PBMC tested on ~ day 15, *n* = 19), third cycle (PBMC tested on ~ day 43, *n* = 14), and ninth cycle (PBMC tested on ~ day 127, *n* = 16) of avelumab. PBMC were counted and assessed for cell viability with trypan blue exclusion. Median cell viability was 92% (89–95% interquartile range), and less than 5% of PBMC samples assayed from the trial (4/81) had viabilities <80%. Samples with viability <80% were annotated in the analysis to ensure that low viability did not impact the ability to detect PD-L1. PBMC were stained with five different antibody panels (Additional file 1: Table S1). These panels identified markers involved in PD-1 signaling (panel 1), CD4^+^ and CD8^+^ T cells, and B cells (panel 2), regulatory T cells (Tregs) (panel 3), NK cells, NK-T cells, conventional dendritic cells (cDC) and plasmacytoid DC (pDC) (panel 4), and myeloid derived suppressor cells (MDSC) (panel 5). A total of 123 peripheral immune cell subsets were analyzed (Table 1 and Additional file 1: Table S2), which included nine classic immune cell subsets, 25 PD-L1^+^ subsets, and 89 refined subsets related to maturation and function. Following staining, samples were acquired on a BD LSRII cytometer (BD Biosciences, San Jose, CA) equipped with four lasers (UV, violet, blue, and red). The cytometer is serviced by BD technicians under a service contract, and is calibrated daily before use with cytometer setup and tracking beads to ensure quality performance. The laser configuration and filter sets are shown in Additional file 2: Figure S1. Voltages were adjusted to allow both negative and positive signals to be visualized and to minimize spectral overlap between channels. FCS files were exported and analyzed using FlowJo v9.7 for Macintosh (Treestar, Ashland, OR) using the outlined gating strategy (Fig. 1), with non-viable cells excluded and negative gates set based on fluorescence minus one controls. Compensation was performed in FlowJo using beads (BD Biosciences) that were single stained with each of the antibodies in the 5 panels. The frequency of all subsets was calculated as a percentage of PBMC to help eliminate the bias that could occur in the smaller populations with fluctuations in leukocyte subpopulations. The methodology used to analyze the flow cytometry data in the current study is identical to that of Lepone et al. [15].

**Table 1:** Complete list of 123 peripheral immune cell subsets analyzed by flow cytometry

1. Total CD4^+^ T cells • PD-L1^+^ CD4 • PD-1^+^ CD4 • EOMES^+^ CD4 • TCR^+^ CD4 • Tbet^+^ CD4 • BATF^+^ CD4 • CTLA-4^+^ CD4 • Tim-3^+^ CD4 • ICOS^+^ CD4 o PD-L1^+^ ICOS^+^ CD4 o PD-1^+^ ICOS^+^ CD4 • Total naïve (CCR7^+^CD45RA^+^) CD4 o PD-L1^+^ naïve CD4 o PD-1^+^ naïve CD4 o CTLA-4^+^ naïve CD4 o Tim-3^+^ naïve CD4 • Total central memory (CCR7^+^ CD45RA^-^) CD4 o PD-L1^+^ CM CD4 o PD-1^+^ CM CD4 o CTLA-4^+^ CM CD4 o Tim-3^+^ CM CD4 • Total effector memory (CCR7^-^ CD45RA^-^) CD4 o PD-L1^+^ EM CD4 o PD-1^+^ EM CD4 o CTLA-4^+^ EM CD4 o Tim-3^+^ EM CD4 • Total EMRA (CCR7^-^CD45RA^+^) CD4 o PD-L1^+^ EMRA CD4 o PD-1^+^ EMRA CD4 o CTLA-4^+^ EMRA CD4 o Tim-3^+^ EMRA CD4 2. Total CD8^+^ T cells • PD-L1^+^ CD8 • PD-1^+^ CD8 • EOMES^+^ CD8 • TCR^+^ CD8 • Tbet^+^ CD8 • BATF^+^ CD8 • CTLA-4^+^ CD8 • Tim-3^+^ CD8 • Total naïve (CCR7^+^CD45RA^+^) CD8 o PD-L1^+^ naïve CD8 o PD-1^+^ naïve CD8 o CTLA-4^+^ naïve CD8 o Tim-3^+^ naïve CD8	• Total central memory (CCR7^+^CD45RA^-^) CD8 o PD-L1^+^ CM CD8 o PD-1^+^ CM CD8 o CTLA-4^+^ CM CD8 o Tim-3^+^ CM CD8 • Total effector memory (CCR7^-^ CD45RA^-^) CD8 o PD-L1^+^ EM CD8 o PD-1^+^ EM CD8 o CTLA-4^+^ EM CD8 o Tim-3^+^ EM CD8 • Total EMRA (CCR7^-^CD45RA^+^) CD8 o PD-L1^+^ EMRA CD8 o PD-1^+^ EMRA CD8 o CTLA-4^+^ EMRA CD8 o Tim-3^+^ EMRA CD8 3. Total Tregs • PD-L1^+^ Tregs • PD-1^+^ Tregs • CTLA-4^+^ Tregs • ICOS^+^ Tregs • CD45RA^+^ Tregs • CD49d^-^ Tregs 4. Total B cells • PD-L1^+^ B cells • PD-1^+^ B cells • CTLA-4^+^ B cells • Tim-3^+^ B cells 5. Total NK • PD-L1^+^ NK • PD-1^+^ NK • Tim-3^+^ NK • Total mature (CD16^+^ CD56^dim^) NK o PD-L1^+^ mature NK o PD-1^+^ mature NK o Tim-3^+^ mature NK • Total functional intermediate (CD16^+^ CD56^br^) NK o PD-L1^+^ functional intermediate NK o PD-1^+^ functional intermediate NK o Tim-3^+^ functional intermediate NK	• Total immature (CD16^-^ CD56^br^) NK o PD-L1^+^ immature NK o PD-1^+^ immature NK o Tim-3^+^ immature NK • Total unconventional (CD16^-^ CD56^dim^) NK o PD-L1^+^ unconventional NK o PD-1^+^ unconventional NK o Tim-3^+^ unconventional NK 6. Total NK-T • PD-L1^+^ NK-T • PD-1^+^ NK-T • Tim-3^+^ NK-T 7. Total cDC • PD-L1^+^ cDC • PD-1^+^ cDC • CD83^+^ cDC • Tim-3^+^ cDC 8. Total pDC • PD-L1^+^ pDC • PD-1^+^ pDC • CD83^+^ pDC • Tim-3^+^ pDC 9. Total MDSC • PD-L1^+^ MDSC • PD-1^+^ MDSC • CD16^+^ MDSC • Total monocytic (CD14^+^ CD15^-^) MDSC o PD-L1^+^ mMDSC o PD-1^+^ mMDSC o CD16^+^ mMDSC • Total granulocytic (CD14^-^ CD15^+^) MDSC o PD-L1^+^ gMDSC o PD-1^+^ gMDSC o CD16^+^ gMDSC • Total lineage negative (CD14^-^ CD15^-^) MDSC o PD-L1^+^ lin neg MDSC o PD-1^+^ lin neg MDSC o CD16^+^ lin neg MDSC

Nine classic subsets were identified as well as 114 refined subsets relating to maturation and function within the classic subsets (Lepone et al., ref. [15])

*BATF* basic leucine zipper transcription factor ATF-like, *cDC* conventional dendritic cells, *CM* central memory, *CTLA-4* cytotoxic T lymphocyte-associated protein-4, *EM* effector memory, *EMRA* terminally differentiated effector memory, *EOMES* eomesodermin, *gMDSC* granulocytic MDSC; *ICOS* inducible T cell co-stimulator, *lin neg MDSC* lineage negative MDSC, *MDSC* myeloid derived suppressor cell, *mMDSC* monocytic MDSC, *NK* natural killer, *pDC* plasmacytoid DC, *PD-1* programmed cell death protein 1, *PD-L1* programmed cell death ligand-1, *Tbet* T box expressed in T cells, *TCR* T cell receptor, *Tim-3* T cell immunoglobulin and mucin domain-3, *Tregs* regulatory T cells

### Measurement of soluble factors in plasma

Plasma levels of sCD27 and sCD40L were determined using human sCD27 and sCD40L Instant ELISA kits (eBioscience, San Diego, CA). One vial of frozen plasma per cancer patient was assayed prior to therapy and following one cycle (~day 15, *n* = 39) and three cycles (~day 43, *n* = 33) of avelumab.

### In vitro ADCC assay

NK effectors were isolated from PBMC using the Human NK Cell Isolation (negative selection) Kit (Miltenyi Biotech, San Diego CA) following the manufacturer’s protocol, resulting in >90% purity. NK cells were allowed to rest overnight in RPMI 1640 medium (Corning Cellgro, Manassas, VA) supplemented with 10% human AB serum (Omega Scientific, Tarzana, CA), 100 U/mL penicillin, 100 μg/mL streptomycin and 2 mM L-glutamine (Corning Cellgro, Corning, NY).

Human lung NCI-H441 [H441] (ATCC® HTB-174™) and NCI-H460 [H460] (ATCC® HTB-177™) carcinoma cells as well as PBMC from healthy donors and cancer patients were used as targets with purified NK cells as effectors in the presence of avelumab or isotype control antibody at a concentration of 1 ng/mL. Prior to use as targets in the ADCC assay, PBMC from cancer patients were sorted to enrich for PD-L1^+^ and PD-L1^-^ cells; PBMC were rested overnight, stained with PD-L1-PE-Cy7 (clone MIH-1, BD Biosciences) followed by Anti-Cy7 microbeads (Miltenyi Biotech) and PD-L1^+^ and PD-L1^-^ fractions isolated by magnetic selection. To determine the ADCC activity, a 4-h ^111^. In-release assay was performed, as previously described [3]. Effector cell-to-target cell (E:T) were used at ratios of 25:1, 12.5:1 and 6.25:1 for assays with healthy donor NK, and 25:1 for experiments with cancer patient NK. Spontaneous release was determined by incubation of target cells with medium alone, and complete lysis by incubation with 0.05% Triton X-100. Specific ADCC lysis was determined using the following equation: percent lysis = (experimental - spontaneous)/(complete - spontaneous) x 100.

### Antibody competition assay

PBMC from a cancer patient with mCRPC were rested for 48 h and then pre-incubated for 30 min with avelumab or IgG1 isotype control at 0.2, 2, and 20 μg/mL, washed and then stained with multiparametric flow cytometry, as described above, to detect PD-L1 (with the MIH-1 clone) within the various immune cell types. PBMC that were not pre-incubated with avelumab or the isotype control also served as controls. The frequency of PD-L1 within each of the classic immune cell types was determined both as a percentage of parent and total PBMC.

### FcγRIIIa (CD16) genotyping

To examine the polymorphism of CD16 (valine (V) versus phenylalanine (F) substitution at amino acid position 158), DNA was extracted from PBMC of healthy donors and cancer patients receiving avelumab using the QIAamp DNA Blood Mini kit (Qiagen, Valencia, CA) and stored at −80 °C until use. The FcγRIIIa (CD16) genotype was determined by performing allele-specific droplet digital polymerase chain reaction (ddPCR) using the TaqMan array for CD16 (rs396991; Life Technologies, Carlsbad, CA) [18]. A master reaction mix was prepared, and 1 μL of DNA was added. The PCR reaction was performed as previously described [3].

### Statistical analyses

Statistical analyses were performed using GraphPad Prism 6 (GraphPad Software, La Jolla, CA), and *p*-values were calculated using the Wilcoxon matched-pairs signed rank test. For the flow cytometry analysis, due to the large number of tests performed, *p*-values were adjusted using Holm’s method (step-down Bonferroni), as previously described [15, 19]. The adjustment was made for the number of subsets with a frequency above 0.01% of PBMC (*n* = 9 for classic subsets, *n* = 29 for subsets in CD4^+^ T cells, *n* = 25 for CD8^+^ T cells, *n* = 5 for Tregs, *n* = 14 for NK cells, *n* = 3 for NK-T cells, *n* = 4 for B cells, *n* = 2 for cDCs, *n* = 3 for pDCs, *n* = 15 for MDSC). Subsets with a potentially biologically relevant change were defined as subsets with a Holm adjusted *p* < 0.05, majority of patients >25% change, difference in medians of pre- vs. post-therapy >0.01% of PBMC, and a frequency >0.01% of PBMC. Trends were defined as subsets with an unadjusted *p* < 0.05, along with the additional criteria used to define changes.

## Results

To interrogate any potential effects of the anti-PD-L1 monoclonal (avelumab) on immune cells in cancer patients, we employed five panels of antibodies in flow cytometry analyses to define 123 different immune cell subsets. The antibody panels used are shown in Additional file 1: Table S1. The complete list of subsets analyzed is shown in Table 1. Additional file 1: Table S2 shows the “classic” immune cell types (CD4^+^ and CD8^+^ T cells, T regulatory cells (Tregs), B cells, NK cells, NK-T cells, cDC, pDC, MDSC) and the various markers used to identify the “refined” subsets within each classic cell type, along with a brief description of what is generally known about each subset. Figure 1 shows an example of the flow cytometry gating strategy employed in these studies using PBMC from a cancer patient following nine cycles of avelumab to identify the 123 immune cell subsets.

As the ability to correctly detect and enumerate PD-L1 positive immune cells is fundamental for this study, experiments were performed to ensure that PD-L1 positive cells could be correctly identified in the staining and gating schema. PBMC from a healthy donor were stained with PD-L1 alone, as well as with multiparametric stains of PD-L1 in each of the five staining panels, and assessed for PD-L1 surface expression, with fluorescence minus one (FMO) controls used for gating. Total PBMC, as well as those in the lymphocyte-monocyte region and non-lymphocyte-monocyte region, expressed a similar frequency of PD-L1 when cells were stained with PD-L1 alone, or PD-L1 in the multiparametric stains, demonstrating that this antibody is compatible in combination with the other antibodies that are needed to identify the various immune cell subsets (Additional file 2: Figure S2). In addition, experiments were undertaken to ensure that the PD-L1 clone used to detect surface PD-L1 in the various immune cell subsets was not blocked by the potential binding of avelumab to the immune cells. PBMC were pre-incubated for 30 min with avelumab or an IgG1 control (0.2, 2, and 20 μg/mL), and then stained with the antibody panels to detect PD-L1 expression in the various immune cell subsets. As seen in Additional file 2: Table S3, PD-L1 was detectable, and measured at a very similar frequency within immune cell subsets (data shown of CD4, CD8, cDC and B cells, both as a percentage of parent and percentage of total PBMC), regardless of whether the PBMC were pre-incubated with the IgG1 isotype control or avelumab. These results demonstrate that the PD-L1 clone (MIH-1) used in the present study to assess surface expression of PD-L1 does not compete for binding with avelumab in PBMC, and can thus be used to measure PD-L1 expression in patients treated with avelumab.

**Fig. 2:**
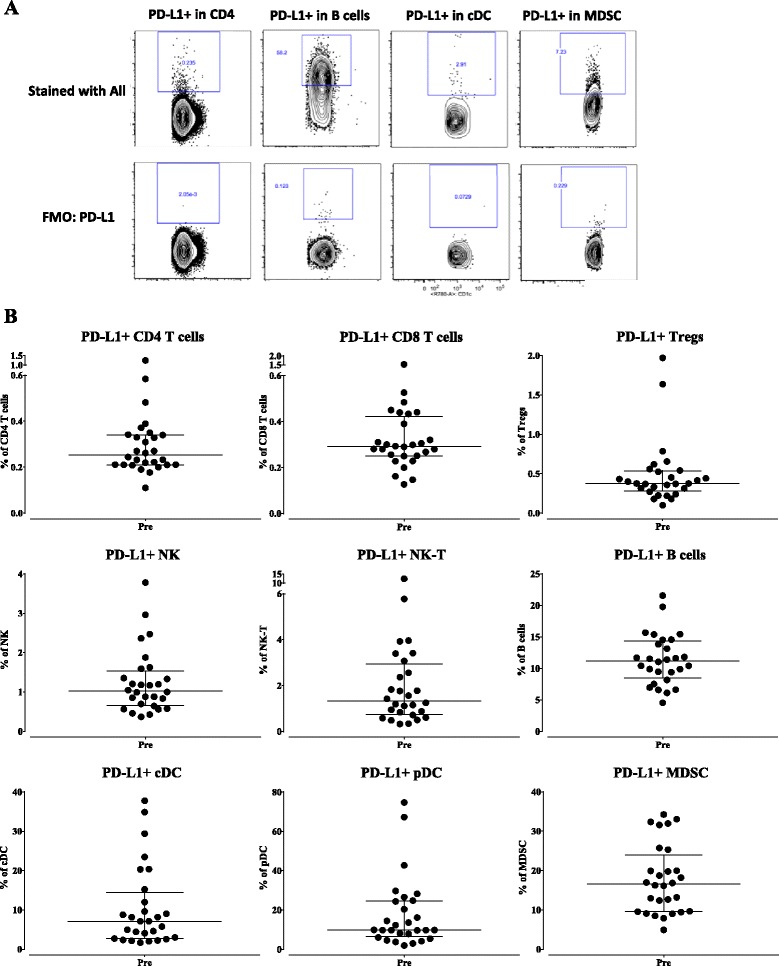
Baseline (pre-treatment) expression of PD-L1 as a percentage of parental classic subset. **a** Representative flow cytometry plots of PD-L1 expression in CD4^+^ T cells, B cells, cDC, and MDSC. **b** In 28 patients prior to avelumab therapy, expression of PD-L1 was measured by flow cytometry for nine classic subsets as a percentage of total PBMC, with graphs displaying median and interquartile range

**Fig. 3:**
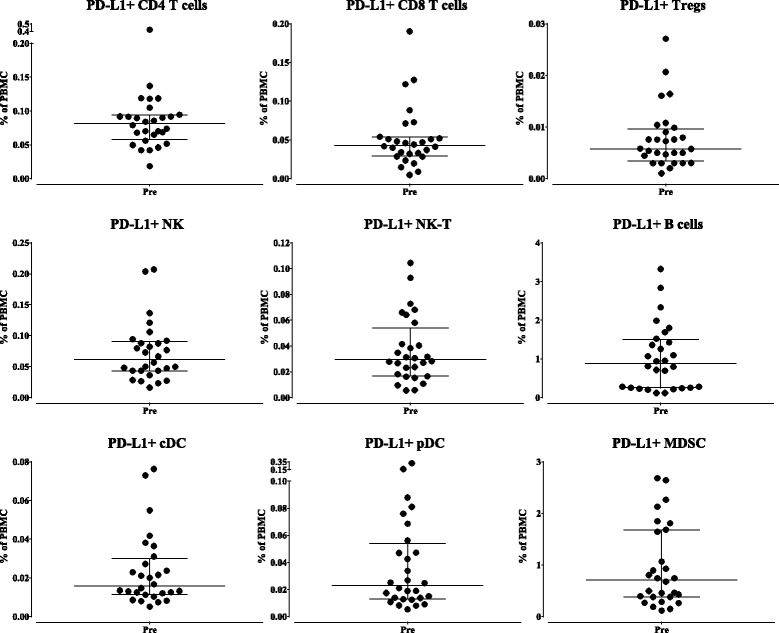
Baseline (pre-treatment) expression of PD-L1 as a percentage of total PBMC. In 28 patients prior to avelumab therapy, expression of PD-L1 was measured by flow cytometry for nine classic subsets as a percentage of total PBMC, with graphs displaying median and interquartile range

PBMC from 28 patients in the Phase I trial were first analyzed at baseline (prior to receiving avelumab) for the surface expression of PD-L1 on each of the nine classic cell types and 16 refined subsets. Table 2 shows the median percentage of each cell type expressing PD-L1, both as a percentage of total PBMC as well as a percentage of the parental cell type. Representative flow cytometry plots of PD-L1 within CD4^+^ T cells, B cells, cDC and MDSC, as well as FMO control used for gating, are shown in Fig. 2a. Figure 2b shows the range at baseline of expression of PD-L1 on the nine classic cell types as a percentage of each classic parental subset, and Fig. 3 shows the range of expression at baseline of PD-L1 of a given cell type as a percentage of total PBMC. For example, while only 0.1 to 3% of PBMC are PD-L1^+^ MDSC (Fig. 3, lower right panel), 5 to 35% of MDSC express PD-L1 (Fig. 2b, lower right panel). Also shown is the variation in PD-L1 expression amongst the parental immune cell types with the highest level of expression of PD-L1 seen on B cells and MDSC (Fig. 3).

**Table 2:** Baseline expression of PD-L1

Classic subsets	Refined subsets	Median % PBMC	Median % parent
PD-L1^+^ CD4		0.08	0.25
	PD-L1^+^ ICOS^+^ CD4	0.05	0.81
	PD-L1^+^ naïve CD4	0.01	0.26
	PD-L1^+^ CM CD4	0.02	0.28
	PD-L1^+^ EM CD4	0.06	0.31
	PD-L1^+^ EMRA CD4	0.01	0.35
PD-L1^+^ CD8		0.04	0.29
	PD-L1^+^ naïve CD8	<0.01	0.40
	PD-L1^+^ CM CD8	<0.01	0.41
	PD-L1^+^ EM CD8	0.02	0.26
	PD-L1^+^ EMRA CD8	0.01	0.30
PD-L1^+^ Tregs		<0.01	0.38
PD-L1^+^ NK		0.06	1.03
	PD-L1^+^ mature NK	0.04	0.97
	PD-L1^+^ functional intermediate NK	<0.01	0.31
	PD-L1^+^ immature NK	<0.01	0.66
	PD-L1^+^ unconventional NK	0.02	5.58
PD-L1^+^ NK-T		0.03	1.34
PD-L1^+^ B cells		0.88	11.22
PD-L^1+^ cDC		0.02	7.12
PD-L1^+^ pDC		0.02	9.85
PD-L1^+^ MDSC		0.71	16.54
	PD-L1^+^ mMDSC	0.10	4.81
	PD-L1^+^ gMDSC	0.26	35.77
	PD-L1^+^ lin neg MDSC	0.17	18.75

In 28 patients prior to avelumab therapy, expression of PD-L1 was measured by flow cytometry in 9 classic subsets and 16 refined subsets as both percentage of total PBMC and of parental cell type

*cDC* conventional dendritic cells, *CM* central memory, *EM* effector memory, *EMRA* terminally differentiated effector memory, *gMDSC* granulocytic MDSC, *ICOS* inducible T cell co-stimulator, *lin neg MDSCs* lineage negative MDSCs, *MDSC* myeloid derived suppressor cell, *mMDSC* monocytic MDSC, *NK* natural killer, *pDC* plasmacytoid DC, *PD-L1* programmed cell death ligand-1, *Tregs* regulatory T cells

**Fig. 4:**
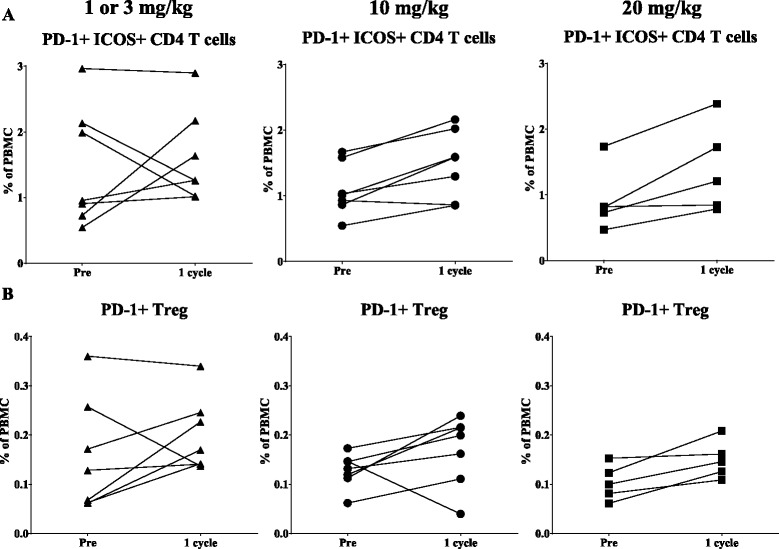
Immune cell subsets of a potentially biologic relevance following different doses of avelumab. Graphs display frequency as a percentage of PBMC for patients treated with 1 or 3 mg/kg (left panels, triangle), 10 mg/kg (middle panels, circle), and 20 mg/kg (right panels, square) of avelumab

Changes in the levels of PD-L1 expressing cells in PBMC were then evaluated after patients received one, three and nine cycles of avelumab every 2 weeks. Using the criteria of a Holm adjusted *p*-value as detailed in the Methods section, there were no significant changes in any of the nine classic immune cell types, or in the PD-L1 expressing classic cell subsets following one, three or nine cycles of avelumab. No differences were seen in results after one or three cycles of avelumab; thus only the results after one cycle and nine cycles are shown; data following three cycles of avelumab is available in Additional file 1: Table S4. Table 3A shows the percentage of patients who had an increase of more than 25%, a minimal change (change less than the 25% cutoff), or a decrease of more than 25% in the nine classic cell types after one or nine cycles of avelumab. There were some trends in classic cell types expressing PD-L1 after nine bi-weekly cycles of avelumab (Table 3B); there was an increase in some patients of PD-L1^+^ pDC and PD-L1^+^ MDSC. None of these trends, however, met statistical significance using the Holm adjusted criteria. We also evaluated additional refined subsets expressing PD-L1 for changes after one, three and nine cycles of avelumab. Only one PD-L1^+^ refined subset met statistical significance using the Holm adjusted criteria, and this change (an increase) only occurred following nine cycles of avelumab (PD-L1^+^ lineage negative MDSC, Holm adjusted *p* = 0.0015) (Additional file 1: Table S5). We also evaluated an additional 89 refined immune cell subsets relating to maturation and function. Table 4 shows the subsets that met the criteria for a trend (unadjusted *p*-value) at any dose; however, none met statistical significance for a change (Holm adjusted *p*-value). Representative graphs show degree of change in subsets meeting the trend criteria in patients receiving one cycle of 1, 3, 10, or 20 mg/kg of avelumab (Fig. 4). Trends in change from baseline were seen in more cell types after nine cycles of avelumab vs. one or three cycles (Table 4). Since these were patients with metastatic disease, these changes could be due to disease progression; however, none were statistically significant using the Holm adjusted *p*-value (Table 4).

**Table 3:** Effect of avelumab on classic and PD-L1^+^ classic immune cell subsets

	Pre vs 1 cycle				Pre vs 9 cycles			
Subset	Increase	Minimal change	Decrease	*P*-value	Increase	Minimal change	Decrease	*P*-value
A. Classic subsets								
CD4	0 (0%)	18 (95%)	1 (5%)	0.0230 (↑^, 0.2070)	0 (0%)	12 (75%)	4 (25%)	0.0063 (↓^, 0.0567)
CD8	1 (5%)	16 (84%)	2 (11%)	0.1447 (=)	3 (19%)	11 (68%)	2 (13%)	0.9799 (=)
Tregs	7 (37%)	10 (53%)	2 (10%)	0.2253 (=)	2 (12%)	7 (44%)	7 (44%)	0.0934 (=)
NK	5 (26%)	10 (53%)	4 (21%)	0.8288 (=)	0 (0%)	9 (56%)	7 (44%)	0.0182 (↓^, 0.1456)
NK-T	2 (11%)	15 (78%)	2 (11%)	0.2413 (=)	1 (6%)	12 (75%)	3 (19%)	0.1046 (=)
B cells	5 (26%)	12 (63%)	2 (11%)	0.7381 (=)	6 (38%)	5 (31%)	5 (31%)	0.8209 (=)
cDC	3 (16%)	15 (79%)	1 (5%)	0.3955 (=)	6 (37%)	7 (44%)	3 (19%)	0.7436 (=)
pDC	6 (32%)	8 (42%)	5 (26%)	0.4900 (=)	6 (38%)	9 (56%)	1 (6%)	0.0833 (=)
MDSC	8 (42%)	8 (42%)	3 (16%)	0.1232 (=)	8 (50%)	7 (44%)	1 (6%)	0.0833 (=)
B. PD-L1^+^ classic subsets								
PD-L1^+^ CD4	5 (26%)	9 (48%)	5 (26%)	0.9843 (=)	3 (19%)	9 (56%)	4 (25%)	0.9999 (=)
PD-L1^+^ CD8	4 (21%)	9 (47%)	6 (32%)	0.1688 (=)	4 (25%)	9 (56%)	3 (19%)	0.9999 (=)
PD-L1^+^ Treg	n/a	n/a	n/a	n/a	n/a	n/a	n/a	n/a
PD-L1^+^ NK	5 (26%)	6 (32%)	8 (42%)	0.4180 (=)	7 (44%)	7 (44%)	2 (12%)	0.1046 (=)
PD-L1^+^ NK-T	5 (26%)	8 (42%)	6 (32%)	0.4900 (=)	6 (38%)	6 (38%)	4 (24%)	0.1439 (=)
PD-L1^+^ B cells	9 (47%)	6 (32%)	4 (21%)	0.6507 (=)	6 (37%)	7 (44%)	3 (19%)	0.5282 (=)
PD-L1^+^cDC	5 (26%)	8 (42%)	6 (32%)	0.3736 (=)	2 (13%)	4 (25%)	10 (62%)	0.0386 (↓*, 0.0772)
PD-L1^+^ pDC	3 (16%)	2 (10%)	14 (74%)	0.0258 (↓*, 0.0774)	10 (63%)	1 (6%)	5 (31%)	0.0443 (↑, 0.1005)
PD-L1^+^ MDSC	8 (42%)	6 (32%)	5 (26%)	0.6507 (=)	10 (63%)	4 (25%)	2 (12%)	0.0131 (↑, 0.1703)

Classic subsets (A) and PD-L1^+^ classic subsets (B) were examined pre-therapy and post-1 cycle (*n* = 19) and 9 cycles (*n* = 16) of avelumab. Results are displayed as the number of patients (percentage of total patients) with an increase of more than 25%, minimal change of less than 25%, and a decrease of more than 25% compared to pre-therapy. Unadjusted *p*-values (= indicates no change; arrows indicate direction of change compared to pre-therapy; and Holm adjusted *p*-value is listed for subsets with significant unadjusted *p*-value) were calculated using the Wilcoxon matched-pairs signed rank test. n/a = not applicable as frequency of subset <0.01% PBMC; ^ = majority of patients with minimal change; * = difference in medians pre- vs post-therapy < 0.01

*cDC* conventional dendritic cells, *MDSC* myeloid derived suppressor cell, *NK* natural killer, *pDC* plasmacytoid DC, *PBMC* peripheral blood mononuclear cell, *Tregs* regulatory T cells

**Table 4:** Effect of avelumab on 89 refined immune cell subsets

Post	Subset	Increase	Minimal change	Decrease	Unadjusted *P*-value	Direction	Holm adjusted *P*-value
1 cycle	PD-1^+^ ICOS^+^ CD4	12 (63%)	5 (26%)	2 (11%)	0.0181	↑	0.5611
	PD-1^+^ Tregs	12 (63%)	5 (26%)	2 (11%)	0.0141	↑	0.0705
	Functional intermediate NK	10 (53%)	6 (31%)	3 (16%)	0.0401	↑	0.5213
3 cycles	Functional intermediate NK	1 (7%)	4 (29%)	9 (64%)	0.0295	↓	0.4130
9 cycles	CTLA-4^+^ EM CD8	9 (56%)	6 (38%)	1 (6%)	0.0250	↑	0.6250
	Functional intermediate NK	2 (12%)	4 (25%)	10 (63%)	0.0386	↓	0.5018
	PD-1^+^ pDC	10 (63%)	2 (12%)	4 (25%)	0.0335	↑	0.1005
	CD16^+^ MDSC	9 (56%)	5 (31%)	2 (13%)	0.0092	↑	0.1288
	gMDSC	11 (69%)	2 (12%)	3 (19%)	0.0131	↑	0.1703
	CD16^+^ gMDSC	10 (62%)	3 (19%)	3 (19%)	0.0155	↑	0.1705
	PD-1^+^ lin neg MDSC	10 (62%)	4 (25%)	2 (13%)	0.0182	↑	0.1820

The frequency of 89 refined immune cell subsets was examined pre-therapy and post-1 cycle (*n*=19), 3 cycles (*n*=14), and 9 cycles (*n*=16) of avelumab. Table displays subsets that met criteria as a potentially biologically relevant trend. Results are displayed as the number of patients (percentage of total patients) with an increase of more than 25%, minimal change of less than 25%, and a decrease of more than 25% compared to pre-therapy. Unadjusted *p*-values (direction of change compared to pre-therapy) were calculated using the Wilcoxon matched-pairs signed rank test, and Holm adjustment was made for the number of subsets within the classic subsets with a frequency above 0.01% of PBMC

*EM* effector memory, *gMDSC* granulocytic MDSC, *ICOS* inducible T cell co-stimulator, *lin neg MDSC* lineage negative MDSC, *MDSC* myeloid derived suppressor cell, *NK* natural killer, *pDC* plasmacytoid DC, *PD-1* programmed cell death protein 1, *Tregs* regulatory T cells

**Fig. 5:**
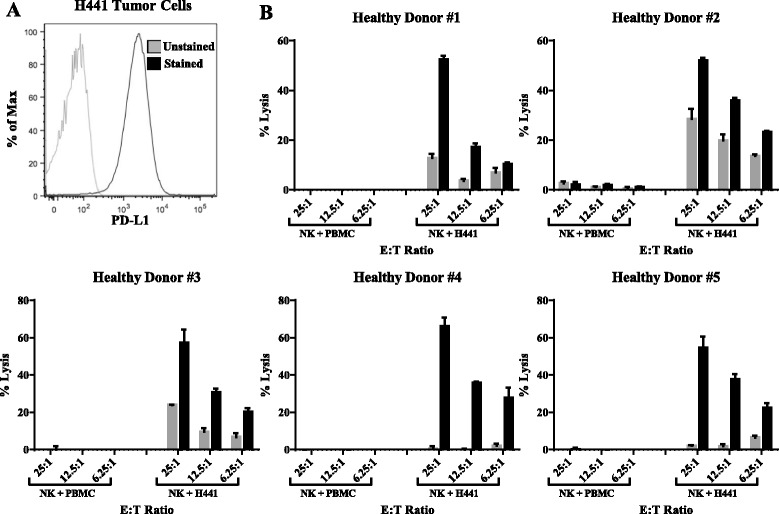
ADCC assay using PBMC from healthy donors or H441 human lung tumor cells as targets. **a** PD-L1 expression in H441 cells. **b** NK cells were purified from PBMC from five healthy donors using negative magnetic selection. In vitro ADCC assays were performed at effector:target ratios of 25:1, 12.5:1, and 6.25:1, using an IgG1 isotype control antibody (gray bars, 1 ng/mL) or avelumab (black bars, 1 ng/mL). Results are displayed as mean + SEM of triplicate wells

We also conducted additional studies using autologous PBMC from healthy donors as targets to determine if avelumab would mediate ADCC of PBMC using NK cells as effectors. We have previously shown [3] that avelumab can mediate ADCC against the human lung cancer line H441; thus it was used as a positive control. As seen in Fig. 5a, 100% of H441 tumor cells express PD-L1, the target of avelumab. Using NK cells isolated from five healthy donors as effector cells, avelumab mediated appreciable ADCC of H441 target cells at multiple effector to target ratios compared to the isotype control MAb (which denotes endogenous NK lysis) (Fig. 5b). Using the same healthy donor NK cells with avelumab, no lysis of autologous PBMC was seen; however, due to the sensitivity of the assay, one cannot rule out the lysis of a minor subpopulation of cells that express PD-L1.

**Fig. 6:**
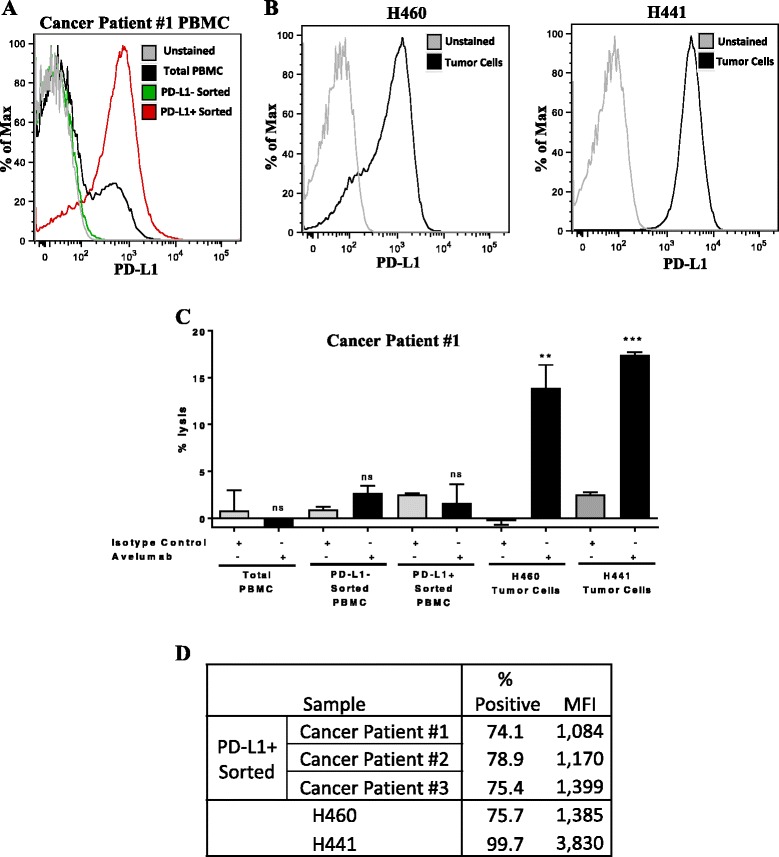
ADCC assay using NK cells from a cancer patient with NSCLC against autologous PBMC sorted to enrich for PD-L1, or H460 or H441 human lung tumor cells. **a** PD-L1 expression in total PBMC, and PBMC magnetically sorted via negative and positive selection to enrich for PD-L1 negative and PD-L1 positive fractions, respectively. **b** PD-L1 expression in H460 and H441 cells. **c** NK cells were purified from PBMC from a cancer patient using negative magnetic selection. In vitro ADCC assays were performed at effector:target ratio of 25:1, using an IgG1 isotype control antibody (gray bars, 1 ng/mL) or avelumab (black bars, 1 ng/mL). Results are displayed as mean + SEM of triplicate wells. Data analyzed with unpaired *T*-test comparing avelumab vs isotype control; **p < 0.01, ***p < 0.001; ns: not significant. **d** Comparison of PD-L1 expression (% positivity and mean fluorescence intensity, MFI) in the PD-L1^+^ enriched PBMC fraction from three cancer patients compared to H460 and H441 cells

Studies were performed to address the concern of the ADCC assay sensitivity, as well as to investigate the ability of cancer patient NK cells to mediate ADCC of PD-L1 expressing targets. NK cells were isolated from a metastatic cancer patient with NSCLC, and tested for their ability to induce avelumab mediated ADCC against autologous PBMC that were sorted to enrich for PD-L1 positive and negative subsets. As seen in Fig. 6a, compared to total PBMC, in which 17% of cells were positive for PD-L1, expression of PD-L1 was negligible (<1% positive) in the PD-L1 negative sorted fraction and markedly enriched in the PD-L1 positive sorted fraction (74% positive). Human lung cancer lines in which 76% of cells expressed PD-L1 (H460) and 100% of cells expressed PD-L1 (H441) were used as positive controls (Fig. 6b). Here, using NK cells from a cancer patient as effector cells, avelumab mediated appreciable ADCC of both H460 and H441 target cells at an effector:target ratio of 25:1 compared to the isotype control MAb; however, using the same patients NK cells with avelumab, no lysis of either total, PD-L1 negative, or PD-L1 positive enriched PBMC was detected (Fig. 6c). Similar results were observed with NK cells from two additional cancer patients (data not shown). Notably, the expression of PD-L1 in the positively sorted fraction of cancer patient PBMC (both % and MFI) was similar to that of the H460 cells, but less than that seen in the H441 cells (Fig. 6d).

**Fig. 7:**
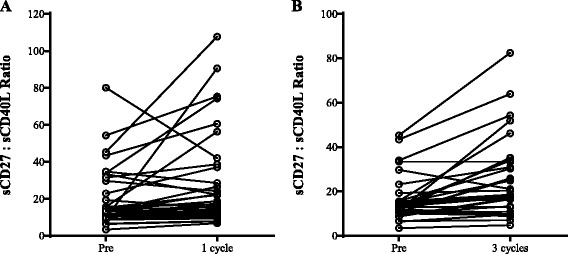
Effect of avelumab on soluble factors. Plasma levels of sCD27 and sCD40L were measured at baseline (pre-therapy) and following one cycle (**a**, *n* = 39) and three cycles (**b**, *n* = 33) of avelumab. Graphs display the ratio of sCD27 to sCD40L. Following one and three cycles of avelumab, there was a statistically significant increase in the ratio (*p* = 0.0087 and *p* = 0.0001, respectively, using the Wilcoxon matched-pairs signed rank test)

As NK CD16 polymorphism has been shown [20] to contribute to the variability of the level of ADCC mediated by NK cells, the genotype of the NK CD16 used as effectors was examined for the healthy donors in these experiments. Four of the five healthy donors used in the in vitro ADCC assay had the V/F genotype, while one had the F/F genotype; the genotype of the cancer patients used in vitro ADCC assays is unknown. The genotypes of 25 of the 28 patients treated with avelumab that were assessed by flow cytometry were determined; 12 patients had the F/F genotype, 11 had the V/F genotype, and 2 had the V/V genotype.

We have previously shown [21, 22] that sCD27 in serum is a marker of T-cell activation and sCD40L in serum is an indicator of immune suppression. Preliminary data has also suggested that increases in the ratio of sCD27:sCD40L correlate with improved patient outcome in some immunotherapy trials. As seen in Fig. 7, there were statistical increases in the sCD27:sCD40L ratio after one administration and three administrations of avelumab (*p* = 0.0087 and *p* = 0.0001, respectively).

## Discussion

The limited toxicity observed in the dose escalation Phase I trial and Phase II trials, and the results described here on the minimal effect of avelumab on 123 immune cell subsets of patients receiving avelumab should help reduce concern about the use of a fully human IgG1 directed against PD-L1. It should be noted that several widely used and effective anti-cancer MAbs, including trastuzumab (Herceptin), cetuximab (Erbitux), and rituximab (Rituxan), are all of the same human IgG1 isotype. While the targets for each of these MAbs are also expressed on some normal tissues, their clinical effectiveness has been shown to outweigh toxicities. The actual mode of action of trastuzumab, cetuximab, and rituximab is generally accepted to be due to the reactivity of each MAb to its respective receptor on tumor cells; evidence also exists, however, that some anti-tumor activity of each of these three antibodies may be due to ADCC. It is known that human NK cells possessing the high affinity V/V CD16 allele generally have more potent ADCC activity than NK cells possessing the lower affinity F/F or V/F CD16 alleles. It has been shown in some prior clinical studies that patients expressing the V/V CD16 allele have greater clinical benefit, when receiving trastuzumab, cetuximab or rituximab, than those patients with the V/F or F/F allele [20, 23]. The genotype of the CD16 allele was assessed in patients of the current Phase I dose escalation trial of avelumab, and the V/V genotype was rarely identified (2/25 patients). In future randomized trials of avelumab, studies will be carried out to determine if clinical benefit of avelumab correlates with the V/V CD16 allele on NK cells.

An anti-PD-L1 MAb of the IgG1 isotype also enables the potential for enhanced ADCC activity by employing agents such as IL-15 and IL-12 immunocytokines. Recent preclinical and clinical studies with recombinant IL-15 [24, 25] and an IL-15/Ra/Fc immunocytokine [26, 27] have shown that these agents greatly enhance the level of NK cells as well as the lytic activity of NK cells on a per cell basis. In vitro studies have shown that IL-12 also enhances NK-mediated ADCC employing avelumab [3]. A tumor targeting IL-12 immunocytokine, designated NHS-IL-12, is currently in clinical studies and may be of use toward this goal. The use in adoptive transfer of allogeneic NK cells (NK-92) has previously been shown to be safe with evidence of clinical activity; a variant of NK-92 genetically engineered to express both IL-2 (and consequent high levels of granzyme) and the high affinity CD16 V/V allele has now been shown to greatly enhance the ADCC activity of avelumab [manuscript submitted for publication]. Since only approximately 10% of humans endogenously express the V/V allele [28], the adoptive transfer of these CD16 high affinity NK (haNK) cells in combination with avelumab merits further investigation.

Analyses of the 123 immune subsets in the periphery also have other potential applications. It has recently been shown [29] that analyses of refined peripheral immune cell subsets in patients from two randomized trials, prior to the initiation of immunotherapy, were able to predict, with statistical significance, those patients more likely to benefit clinically. This was not the case when classic immune cell subsets were analyzed in an identical manner. Results from these two randomized trials showed that the analyses of refined cell subsets did not predict patient benefit in the arms of the trials in which patients received chemotherapy alone or radionuclide alone, but only predicted benefit in the arms of the trials in which patients received vaccine plus chemotherapy or vaccine plus radionuclide. The analyses of immune cell subsets in the periphery, prior to the initiation of immunotherapy, have also been reported by several groups to be a potential indicator of those patients most likely to achieve clinical benefit [30, 31]. While it is clear that analysis of analysis of biopsies for the presence of immune infiltrate has been shown in some cases to be an important predictor of clinical benefit, especially in the analyses of biopsies of primary lesions of colorectal cancer patients [32], the metastatic lesions of many cancer types are not always amenable to biopsy. Moreover, it is known that there is often an interplay between different immune cell types. The analyses of the 123 immune cell subsets in the periphery could add potentially valuable information to that obtained in analyses of available biopsy specimens; thus the dual analyses of specific immune subsets in tumor and in the periphery may be of optimal benefit in helping to define patient outcome. Assessment of soluble factors in sera or plasma that can easily be tracked in patients over time can also provide an overall indication of the immune environment induced following a given therapy. In the present study, exposure to avelumab significantly increased the ratio of sCD27/sCD40L. Previous studies [21, 22] have found that sCD27 is a marker of T-cell activation, while sCD40L is a measure of immune suppression, and that increases and decreases, respectively in these measures in patient serum/plasma, correlate with improved outcome in some immunotherapy trials. Future studies of avelumab in expansion cohorts will assess if there is any link between clinical efficacy and the trends of changes in soluble factors (increase in the ratio of sCD27/sCD40L) and specific peripheral immune cell subsets.

It is understood that there are a number of limitations in the current study. PD-L1, for example, is expressed at a low level in many immune cell types in the periphery; however, as seen in Fig. 2b, there are some subsets such as dendritic cells, B cells, and MDSCs that were identified as expressing higher levels of this marker. In addition, it has been reported that cryopreservation may impact PD-L1 expression, reducing expression on monocytes and CD8^+^ T cells, but not on CD4^+^ T cells [33]. However, because of the need to analyze clinical trial samples in batches (pre- and all post-treatment samples from the same patient assayed at the same time) to reduce other experimental variables, the present study assessed cryopreserved material. Importantly, PBMC samples pre- and post-treatment were isolated and cryopreserved in the same manner following stringent protocols, and a live/dead discriminator was used to exclude dead cells that bind non-specifically to antibody. It is also recognized that PD-L1 expression can be temporally influenced by therapies that induce immune activation, and that expression of immune cells in the periphery may likely differ from what is seen within the tumor. Despite all of these limitations, we show that there is no marked loss of PD-L1 expressing immune cells in the peripheral blood of patients treated with avelumab. Studies are planned to examine the immune effects of avelumab within the tumor microenvironment in future expansion cohorts of avelumab in patients with tumors that can be biopsied, where PD-L1 may be more broadly expressed and upregulated on immune cells by the immune potentiating properties of avelumab.

NK cells are known to express numerous Ig-like receptors and C-type lectin receptors that deliver a finely tuned balance of activating and inhibitory signals that can influence their ability to discriminate between self/ healthy cells from transformed/pathogen infected cells [34]. We demonstrate in the present study that NK cells isolated from metastatic NSCLC patients induce avelumab mediated ADCC of human lung tumor cell lines but not of autologous PBMC, including those that have been sorted to enrich for expression of PD-L1, the target of avelumab. This is in concordance with the finding of Voskens et al. [35], that ex vivo expanded human NK cells expressing high levels of activating receptors can mediate cytotoxicity of cancer cell lines by direct recognition and ADCC, but do not lyse autologous PBMC. In that study it was postulated that the inhibitory ligands expressed on normal PBMC may predominate over activating signals, in order to be able to control the NK-mediated cytolytic activity against non-malignant cells.

## Conclusions

In conclusion, our data show a lack of any significant effect on 123 peripheral immune cell subsets, including those that express PD-L1, following treatment of cancer patients with multiple cycles of avelumab. We also demonstrate in controlled in vitro experiments that avelumab can mediate ADCC of PD-L1 expressing tumor cells, but not against PD-L1 expressing immune cells. These findings complement the limited toxicity observed in the dose escalation Phase I and Phase II trials of avelumab, and should help to reduce concern about the use of a fully human IgG1 MAb directed against PD-L1. This study also supports the rationale to further exploit the potential ADCC mechanism of action of avelumab against tumor cells to potentially enhance clinical activity.

## Additional files

Additional file 1: Table S1. Antibodies used for 5 panel stain to identify 123 peripheral immune cell subsets. Panel 1: PD-1 signaling; Panel 2: CD4^+^ T cells, CD8^+^ T cells, B cells; Panel 3: Tregs; Panel 4: NK, NK-T, cDC, pDC; Panel 5: MDSC. **Table S2.** Peripheral immune cell subsets analyzed by flow cytometry. Analysis of 123 subsets using 30 unique markers. **Table S3.** PD-L1 clone MIH-1 used to detect surface expression of PD-L1 in immune cell subsets does not compete for binding with avelumab. **Table S4.** Effect of three cycles of avelumab on classic and PD-L1^+^ classic immune cell subsets. Classic and PD-L1^+^ classic subsets were examined pre-therapy and post-three cycles (*n* = 14) of avelumab. **Table S5.** Effect of avelumab on PD-L1^+^ refined immune cell subsets. PD-L1^+^ refined subsets were examined pre-therapy and post-one cycle (*n* = 19), three cycles (*n* = 14), and nine cycles (*n* = 16) of avelumab. (PDF 464 kb)
Additional file 2: Figure S1. Configuration of LSR II to allow for the detection of up to 11 markers per staining panel. **Figure S2.** PD-L1 expression in PBMC from a healthy donor stained with single and multiparametric stains of PD-L1. (PDF 520 kb)

## Abbreviations

ADCC: Antibody-dependent cell-mediated cytotoxicity
cDC: Conventional dendritic cells
ddPCR: Droplet digital polymerase chain reaction
DLT: Dose-limiting toxicities
F: Phenylalanine
FMO: Fluorescence minus one
MAb: Monoclonal antibody
mCRPC: Metastatic castration resistant prostate cancer
MDSC: Myeloid derived suppressor cells
MFI: Mean fluorescence intensity
NK: Natural killer
NSCLC: Non-small cell lung cancer
PBMC: Peripheral blood mononuclear cells
PD-1: Programmed cell death protein 1
pDC: Plasmacytoid dendritic cells
PD-L1: Programmed cell death protein-1 ligand
sCD27: Soluble CD27
sCD40L: Soluble CD40 ligand
Tregs: Regulatory T cells
V: Valine
