# Supplementary material for: Analyses of the peripheral immunome following multiple administrations of avelumab, a human IgG1 anti-PD-L1 monoclonal antibody
Source: J Immunother Cancer. 2017 Feb 21;5:20. doi: 10.1186/s40425-017-0220-y (PMC5320726; doi:10.1186/s40425-017-0220-y)
Supplement: Additional file 1: Table S1. — Antibodies used for 5 panel stain to identify 123 peripheral immune cell subsets. Panel 1: PD-1 signaling; Panel 2: CD4+ T cells, CD8+ T cells, B cells; Panel 3: Tregs; Panel 4: NK, NK-T, cDC, pDC; Panel 5: MDSC. Table S2. Peripheral immune cell subsets analyzed by flow cytometry. Analysis of 123 subsets using 30 unique markers. Table S3. PD-L1 clone MIH-1 used to detect surface expression of PD-L1 in immune cell subsets does not compete for binding with avelumab. Table S4. Effect of three cycles of avelumab on classic and PD-L1+ classic immune cell subsets. Classic and PD-L1+ classic subsets were examined pre-therapy and post-three cycles (n = 14) of avelumab. Table S5. Effect of avelumab on PD-L1+ refined immune cell subsets. PD-L1+ refined subsets were examined pre-therapy and post-one cycle (n = 19), three cycles (n = 14), and nine cycles (n = 16) of avelumab. (PDF 464 kb) [file 40425_2017_220_MOESM1_ESM.pdf]

Additional file 1: Table S1. Antibodies used for 5 panel stain to identify 123 peripheral immune cell subsets. Panel 1: PD-1 signaling; Panel 2: $CD4^+$ T cells, $CD8^+$ T cells, B cells; Panel 3: Tregs; Panel 4: NK, NK-T, cDC, pDC; Panel 5: MDSC [15]. Intracellular antibodies are underlined, and clones and company are listed under each antibody.

Fluorochrome	Panel 1	Panel 2	Panel 3	Panel 4	Panel 5
**FITC**	–	CTLA-4 (A3.4H2.H12, LS Bio)	CTLA-4 (A3.4H2.H12, LS Bio)	CD3 (HIT3a, BD)	CD15 (HI98, Ebioscience)
**PE**	PD-1 (MIH4, BD)	PD-1 (MIH4, BD)	PD-1 (MIH4, BD)	PD-1 (MIH4, BD)	PD-1 (MIH4, BD)
**PerCP-Cy5.5**	<u>EOMES</u> (WD1928, Ebioscience)	CCR7 (150503, BD)	ICOS (C398.4A, Biolegend)	CD303 (201A, Biolegend)	–
**PECy7**	PD-L1 (MIH1, BD)	PD-L1 (MIH1, BD)	PD-L1 (MIH1, BD)	PD-L1 (MIH1, BD)	PD-L1 (MIH1, BD)
**BV421**	TCR (IP26, Biolegend)	Tim-3 (F38-2E2, Biolegend)	<u>FoxP3</u> (206D, Biolegend)	Tim-3 (F38-2E2, Biolegend)	CD14 (MOP9, BD)
**V500**	CD4 (OKT4, Biolegend)	CD19 (HIB19, Biolegend)	CD49d (9F10, Biolegend)	CD83 (HB15e, BD)	CD16 (3G8, Biolegend)
**BV605**	<u>Tbet</u> (4B10, Biolegend)	CD4 (RPA-T4, BD)	CD4 (RPA-T4, BD)	CD56 (HCD56, Biolegend)	HLA DR (L243, Biolegend)
**Dapi**	Live/Dead (Invitrogen)	Live/Dead (Invitrogen)	Live/Dead (Invitrogen)	Live/Dead (Invitrogen)	Live/Dead (Invitrogen)
**APC**	<u>BATF</u> (MBM7C7, Ebioscience)	–	CD25 (M-A251, Biolegend)	–	CD33 (WM53, Biolegend)
**AF700**	–	CD45RA (HI100, BD)	CD45RA (HI100, BD)	CD16 (3G8, Biolegend)	–
**APC Cy7**	CD8 (RPA-T8, Biolegend)	CD8 (RPA-T8, Biolegend)	CD127 (eBioRDR5, Ebioscience)	CD1c (L161, Biolegend)	CD11b (ICRF44, BD)

BATF, basic leucine zipper transcription factor ATF-like; cDC, conventional dendritic cells; CTLA-4, cytotoxic T lymphocyte-associated protein-4; EOMES, eomesodermin; FoxP3, forkhead box P3; HLA, human leukocyte antigen; ICOS, inducible T cell co-stimulator; MDSC, myeloid derived suppressor cells; NK, natural killer; pDC, plasmacytoid DC; PD-1, programmed cell death-1; PD-L1, programmed cell death ligand-1; Tbet, T box expressed in T cells; TCR, T cell receptor; Tim-3, T cell immunoglobulin and mucin domain-3; Tregs, regulatory T cells.

Additional file 1: Table S2. Peripheral immune cell subsets analyzed by flow cytometry.
Analysis of 123 subsets using 30 unique markers [Lepone, et al. Analyses of 123 peripheral human immune cell subsets: defining differences with age and between healthy donors and cancer patients not detected in analysis of standard immune cell types. J Circ Biomark. 2016;5].

1. **$CD4^+$ T cells**: Helper T lymphocytes (32 subsets)
2. **$CD8^+$ T cells**: Cytotoxic T lymphocytes (29 subsets)
    - **Markers of PD-1 pathway and T cell activation (in CD4 and CD8):**
        - **EOMES:** activation
        - **TCR:** activation
        - **Tbet:** activation
        - **BATF:** activation/exhaustion
    - **Maturation status of T cells (in CD4 and CD8)**:
        - **Naïve**: $CD45RA^+$ $CCR7^+$
        - **Central Memory (CM)**: $CD45RA^-$ $CCR7^+$
        - **Effector Memory (EM)**: $CD45RA^-$ $CCR7^-$
        - **Terminal (EMRA)**: $CD45RA^+$ $CCR7^-$
    - **T cell markers (in CD4 and CD8)**:
        - **CTLA-4:** inhibition
        - **PD-1:** activation/inhibition
        - **PD-L1:** activation/cross-inhibition
        - **Tim-3:** inhibition
        - **ICOS**: activation (only on CD4)
3. **Tregs**: Regulatory T lymphocytes ($CD4^+$ $CD25^+$ $FoxP3^+$ $CD127^-$) (7 subsets)
    - **CD45RA**: Tregs highly expandable *in vitro*
    - **CTLA-4**: Treg suppression
    - **$CD49d^-$:** suppressive Tregs
    - **ICOS**: Treg suppression
    - **PD-1:** activation/inhibition
    - **PD-L1:** cross-inhibition
4. **B cells**: $CD19^+$ (5 subsets)
    - **CTLA-4:** inhibition
    - **Tim-3:** inhibition
    - **PD-1:** activation/inhibition
    - **PD-L1:** cross-inhibition
5. **NK**: Natural killer cells ($CD56^+$ $CD3^-$) (20 subsets)
    - **$CD16^+$ $CD56^{dim}$**: Mature, lytic
    - **$CD16^+$ $CD56^{br}$**: Functional intermediate, lytic and cytokine production
    - **$CD16^-$ $CD56^{br}$:** Immature, cytokine production, abundant in placenta
    - **$CD16^-$ $CD56^{dim}$**: Unconventional, non-lytic, non-cytokine production
    - **Tim-3:** activation
    - **PD-1:** activation/inhibition
    - **PD-L1:** cross-inhibition
6. **NK-T**: $CD56^+$ $CD3^+$ (4 subsets)
    - **Tim-3:** activation
    - **PD-1:** activation/inhibition
    - **PD-L1:** cross-inhibition
7. **cDCs:** conventional dendritic cells (DCs) ($CD3^-CD56^-CD1c^+CD303^-$) (5 subsets)
8. **pDCs:** plasmacytoid DCs ($CD3^-CD56^-CD1c^-CD303^+$) (5 subsets)
    - **Markers of DC activation**
        - **CD83:** activation
        - **Tim-3:** inhibition
        - **PD-1:** activation/inhibition
        - **PD-L1:** cross-inhibition
9. **MDSCs**: Myeloid derived suppressor cells ($CD11b^+$ $HLA\text{-}DR^{low/-}$ $CD33^+$) (16 subsets)
    - **CD14**: common myeloid marker
    - **CD15**: granulocyte marker
    - **CD16**: immature MDSCs
    - **PD-1:** activation/inhibition
    - **PD-L1:** cross-inhibition

Frozen PBMC were thawed then stained using 30 unique markers in 5 immune flow cytometry panels to identify a total of 123 peripheral immune cell subsets. Samples were collected on an LSR II flow cytometer equipped with UV, red, blue, and violet lasers, and analyzed using FlowJo with gating set using fluorescence minus one controls. Nine classic immune cell types as well as 114 refined subsets relating to maturation and function were examined.

BATF, basic leucine zipper transcription factor ATF-like; CTLA-4, cytotoxic T lymphocyte-associated protein-4; EOMES, eomesodermin; FoxP3, forkhead box P3; HLA, human leukocyte antigen; ICOS, inducible T cell co-stimulator; PBMCs, peripheral blood mononuclear cells; PD-1, programmed cell death-1; PD-L1, programmed cell death ligand-1; Tbet, T box expressed in T cells; TCR, T cell receptor; Tim-3, T cell immunoglobulin and mucin domain-3.

Additional file 1: Table S3. PD-L1 clone MIH-1 used to detect surface expression of PD-L1 in immune cell subsets does not compete for binding with avelumab. PBMC isolated from a patient with metastatic prostate cancer were rested for 48 hours, and then pre-incubated for 30 minutes with the indicated concentrations of avelumab or IgG1 isotype control prior to multiparametric stains of PD-L1 to detect PD-L1 within the various immune cell types. Cells that were not pre-incubated with the isotype control or avelumab also served as controls. PD-L1 was detectable, and measured at a very similar frequency within immune cell subsets (data shown of CD4, CD8, cDC and B cells, both as a percentage of parent and percentage of total PBMC), regardless of whether the PBMC were pre-incubated with the IgG1 isotype control or avelumab. These results demonstrate that the PD-L1 clone (MIH-1) used in the present study to assess surface expression of PD-L1 does not compete for binding with avelumab in PBMC, and can thus be used to measure PD-L1 expression in patients treated with avelumab.

	% of Parent							% of Total PBMC						
**Subset**	**Not Pre-Incubated**	**Pre-Incubated with IgG1 Isotype Control (ug/mL)**			**Pre-Incubated with Avelumab (ug/mL)**			**Not Pre-Incubated**	**Pre-Incubated with IgG1 Isotype Control (ug/mL)**			**Pre-Incubated with Avelumab (ug/mL)**		
		**0.2**	**2**	**20**	**0.2**	**2**	**20**		**0.2**	**2**	**20**	**0.2**	**2**	**20**
**PDL1+ CD4**	0.3	0.3	0.2	0.3	0.2	0.2	0.2	0.09	0.09	0.08	0.10	0.07	0.06	0.07
**PDL1+ CD8**	0.3	0.2	0.2	0.2	0.2	0.2	0.2	0.07	0.06	0.06	0.06	0.06	0.05	0.07
**PDL1+ cDC**	77	64	75	71	66	85	72	0.05	0.02	0.04	0.04	0.04	0.07	0.04
**PDL1+ B cells**	46	44	40	44	46	46	45	2.89	2.97	2.62	2.86	3.08	3.04	2.94

Additional file 1: Table S4. Effect of 3 cycles of avelumab on classic and PD-L1+ classic immune cell subsets. Classic subsets (**A**) and PD-L1+ classic subsets (**B**) were examined pre-therapy and post-3 cycles (n=14) of avelumab. Results are displayed as the number of patients (percentage of total patients) with an increase of more than 25%, minimal change of less than 25%, and a decrease of more than 25% compared to pre-therapy. Unadjusted p-values (= indicates no change; arrows indicate direction of change compared to pre-therapy; and holm adjusted p-value is listed for subsets with significant unadjusted p-value) were calculated using the Wilcoxon matched-pairs signed rank test. n/a = not applicable as frequency of subset <0.01% PBMC; ^ = majority of patients with minimal change. cDC, conventional dendritic cells; MDSC, myeloid derived suppressor cell; NK, natural killer; pDC, plasmacytoid DC; PBMC, peripheral blood mononuclear cell; Tregs, regulatory T cells.

A.

	**Pre vs 3 cycles**			
**Classic Subsets**	**Increase**	**Minimal Change**	**Decrease**	**P-value**
CD4	0 (0%)	14 (100%)	0 (0%)	0.2412 (=)
CD8	0 (0%)	13 (93%)	1 (7%)	0.0580 (=)
Treg	4 (29%)	8 (57%)	2 (14%)	0.5016 (=)
NK	1 (7%)	7 (50%)	6 (43%)	0.0785 (=)
NK-T	0 (0%)	12 (86%)	2 (14%)	0.1189 (=)
B cells	3 (21%)	11 (79%)	0 (0%)	0.5016 (=)
cDC	1 (7%)	9 (64%)	4 (29%)	0.1531 (=)
pDC	4 (29%)	7 (50%)	3 (21%)	0.9032 (=)
MDSC	7 (50%)	4 (29%)	3 (21%)	0.1353 (=)

B.

	**Pre vs 3 cycles**			
**PD-L1+ Classic subsets**	**Increase**	**Minimal Change**	**Decrease**	**P-value**
PDL1+ CD4	1 (7%)	8 (57%)	5 (36%)	**0.0245** **(↓^, 0.7105)**
PDL1+ CD8	0 (0%)	10 (71%)	4 (29%)	0.0785 (=)
PDL1+ Treg	n/a	n/a	n/a	n/a
PDL1+ NK	0 (0%)	10 (71%)	4 (29%)	0.0580 (=)
PDL1+ NK-T	4 (29%)	7 (50%)	3 (21%)	0.4263 (=)
PDL1+ B cells	3 (21%)	9 (65%)	2 (14%)	0.9515 (=)
PDL1+ cDC	7 (50%)	5 (36%)	2 (14%)	0.0785 (=)
PDL1+ pDC	6 (43%)	3 (21%)	5 (36%)	0.7609 (=)
PDL1+ MDSC	7 (50%)	5 (36%)	2 (14%)	0.3575 (=)

Additional file 1: Table S5. Effect of avelumab on PD-L1+ refined immune cell subsets. PD-L1+ refined subsets were examined pre-therapy and post-1 cycle (n=19), 3 cycles (n=14) and 9 cycles (n=16) of avelumab. Results are displayed as the number of patients (percentage of total patients) with an increase of more than 25%, minimal change of less than 25%, and a decrease of more than 25% compared to pre-therapy. Unadjusted p-values (= indicates no change; arrow indicates direction of change compared to pre-therapy; and holm adjusted p-value is listed for subsets with significant unadjusted p-value) were calculated using the Wilcoxon matched-pairs signed rank test. n/a = not applicable as frequency of subset <0.01% PBMC. ^ = majority of patients with minimal change; * = difference in medians pre- vs post-therapy < 0.01.

	**Pre vs 1 cycle**				**Pre vs 3 cycles**				**Pre vs 9 cycles**			
**Subset**	**Increase**	**Minimal Change**	**Decrease**	**P-value**	**Increase**	**Minimal Change**	**Decrease**	**P-value**	**Increase**	**Minimal Change**	**Decrease**	**P-value**
**PD-L1+ ICOS+ CD4**	5 (26%)	11 (58%)	3 (16%)	0.7983 (=)	4 (29%)	6 (42%)	4 (29%)	0.3910 (=)	3 (19%)	9 (56%)	4 (25%)	0.3894 (=)
**PDL1+ naïve CD4**	8 (42%)	6 (32%)	5 (26%)	0.2935 (=)	4 (28%)	5 (36%)	5 (36%)	0.8077 (=)	3 (19%)	9 (56%)	4 (25%)	0.9709 (=)
**PDL1+ CM CD4**	4 (21%)	8 (42%)	7 (37%)	0.3955 (=)	1 (7%)	5 (36%)	8 (57%)	**0.0040** (↓*, 0.1240)	2 (13%)	9 (56%)	5 (31%)	0.1353 (=)
**PDL1+ EM CD4**	6 (32%)	7 (36%)	6 (32%)	0.8288 (=)	2 (14%)	7 (50%)	5 (36%)	**0.0295** (↓^, 0.8260)	5 (31%)	7 (44%)	4 (25%)	0.9901 (=)
**PDL1+ EMRA CD4**	6 (32%)	7 (36%)	6 (32%)	0.7086 (=)	3 (21%)	5 (36%)	6 (43%)	0.4263 (=)	8 (50%)	4 (25%)	4 (25%)	0.1711 (=)
**PDL1+ naïve CD8**	n/a	n/a	n/a	n/a	n/a	n/a	n/a	n/a	n/a	n/a	n/a	n/a
**PDL1+ CM CD8**	n/a	n/a	n/a	n/a	n/a	n/a	n/a	n/a	n/a	n/a	n/a	n/a
**PDL1+ EM CD8**	4 (21%)	10 (53%)	5 (26%)	0.2753 (=)	0 (0%)	9 (64%)	5 (36%)	0.0785 (=)	4 (25%)	9 (56%)	3 (19%)	0.8358 (=)
**PDL1+ EMRA CD8**	3 (16%)	8 (42%)	8 (42%)	0.1232 (=)	1 (7%)	7 (50%)	6 (43%)	0.0785 (=)	7 (44%)	7 (44%)	2 (12%)	0.0975 (=)
**PDL1+ mature NK**	4 (21%)	7 (37%)	8 (42%)	0.3124 (=)	3 (21%)	5 (36%)	6 (43%)	0.4631 (=)	8 (50%)	6 (38%)	2 (12%)	0.1297 (=)
**PDL1+ functional intermediate NK**	n/a	n/a	n/a	n/a	n/a	n/a	n/a	n/a	n/a	n/a	n/a	n/a
**PDL1+ immature NK**	n/a	n/a	n/a	n/a	n/a	n/a	n/a	n/a	n/a	n/a	n/a	n/a
**PDL1+ un-conventional NK**	5 (26%)	10 (53%)	4 (21%)	0.5949 (=)	4 (28%)	5 (36%)	5 (36%)	0.4263 (=)	8 (50%)	5 (31%)	3 (19%)	0.3484 (=)
**PDL1+ mMDSC**	11 (58%)	3 (16%)	5 (26%)	0.3736 (=)	5 (36%)	2 (14%)	7 (50%)	0.6257 (=)	5 (31%)	3 (19%)	8 (50%)	0.4954 (=)
**PDL1+ gMDSC**	3 (16%)	2 (10%)	14 (74%)	0.1447 (=)	9 (65%)	2 (14%)	3 (21%)	0.2676 (=)	10 (62%)	4 (25%)	2 (13%)	0.0833 (=)
**PDL1+ lin neg MDSC**	6 (32%)	7 (36%)	6 (32%)	0.7086 (=)	5 (36%)	6 (43%)	3 (21%)	0.7609 (=)	14 (87%)	2 (13%)	0 (0%)	**0.0001 (↑, 0.0015)**
