# Supplementary material for: Analyses of the peripheral immunome following multiple administrations of avelumab, a human IgG1 anti-PD-L1 monoclonal antibody
Source: J Immunother Cancer. 2017 Feb 21;5:20. doi: 10.1186/s40425-017-0220-y (PMC5320726; doi:10.1186/s40425-017-0220-y)

Additional file 2: Figure S1. Configuration of LSR II. The LSR II used to acquire the stained samples was equipped with four lasers (UV, violet, blue, red) to allow for the detection of up to 11 markers per staining panel. (Lepone et al., ref. 15).

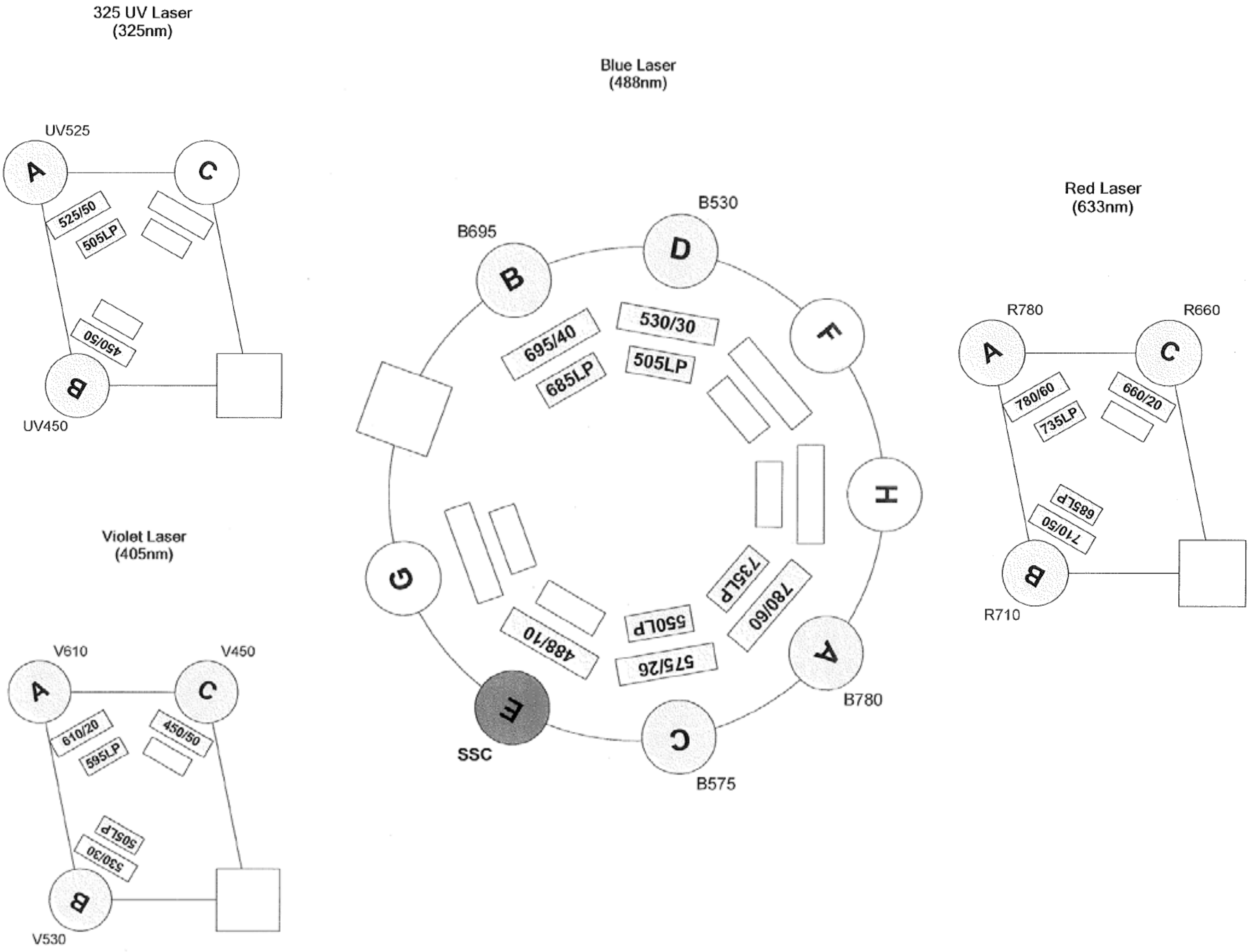

Additional file 2: Figure S2. PD-L1 expression in PBMC from a healthy donor stained with single and multiparametric stains of PD-L1. PBMC were assessed for PD-L1 surface expression vs SSC following single and multi-parametric stains of PD-L1. Following the gating strategy of Figure 1, the lymphocyte-monocyte region was assessed in Panels 1, 2 and 3, while both the lymphocyte-monocyte, and non-lymphocyte-monocyte regions were measured in panels 4 and 5. Fluorescent minus one (FMO) controls were used for gating the multiparametric stained samples. PBMC expressed a similar frequency of PD-L1 following single or multiparametric stains of PD-L1, demonstrating that this antibody is compatible with other antibodies required to identify the various cell types in the multiparametric stains.

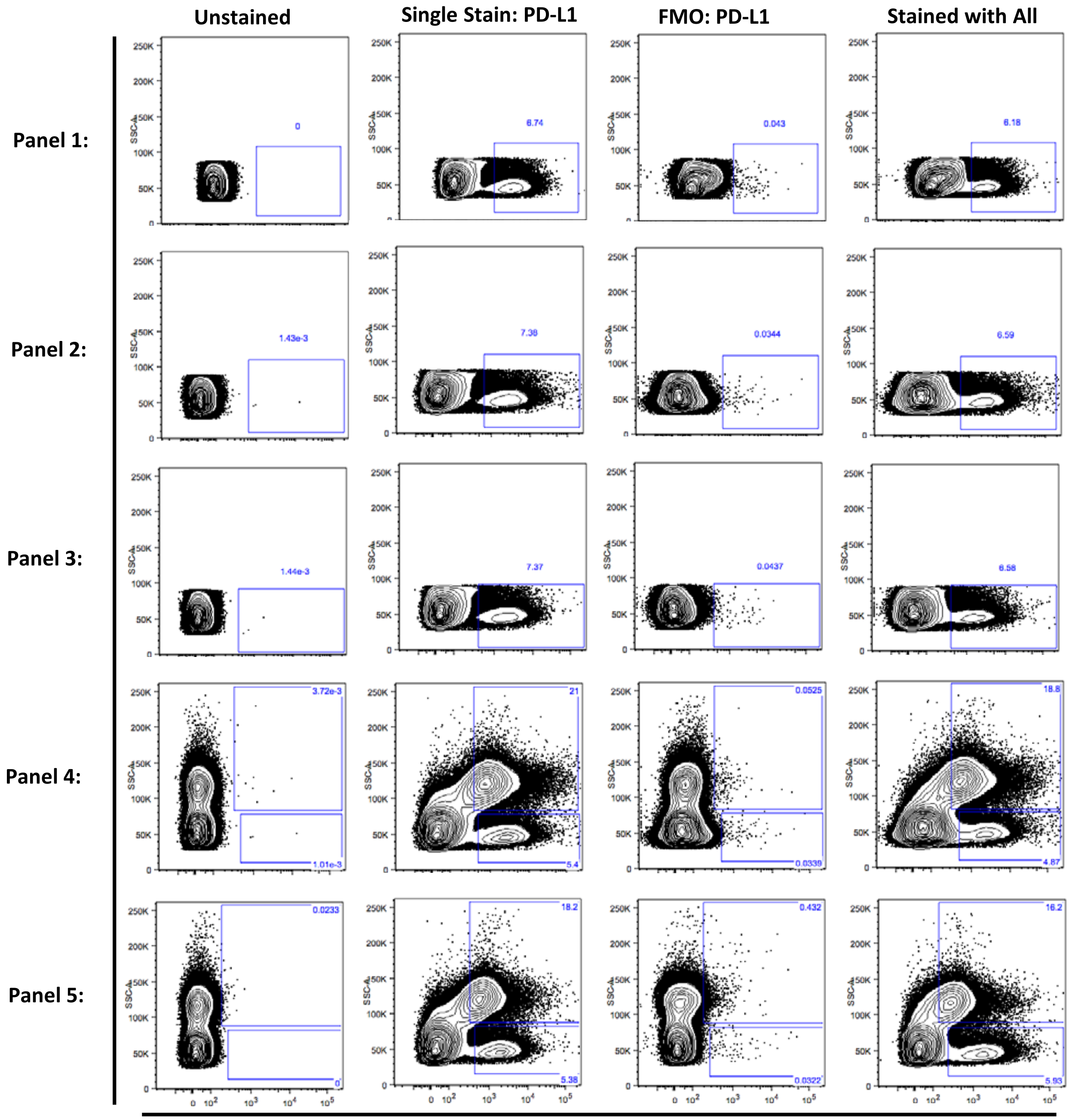

Supplement: Additional file 2: Figure S1. — Configuration of LSR II to allow for the detection of up to 11 markers per staining panel. Figure S2. PD-L1 expression in PBMC from a healthy donor stained with single and multiparametric stains of PD-L1. (PDF 520 kb) [file 40425_2017_220_MOESM2_ESM.pdf]
